# Assessing Cognitive Change and Quality of Life 12 Months After Epilepsy Surgery—Development and Application of Reliable Change Indices and Standardized Regression-Based Change Norms for a Neuropsychological Test Battery in the German Language

**DOI:** 10.3389/fpsyg.2020.582836

**Published:** 2020-10-15

**Authors:** Nadine Conradi, Marion Behrens, Anke M. Hermsen, Tabitha Kannemann, Nina Merkel, Annika Schuster, Thomas M. Freiman, Adam Strzelczyk, Felix Rosenow

**Affiliations:** ^1^Epilepsy Center Frankfurt Rhine-Main, Department of Neurology, University Hospital Frankfurt and Goethe University, Frankfurt, Germany; ^2^LOEWE Center for Personalized Translational Epilepsy Research, Goethe University, Frankfurt, Germany; ^3^Department of Neurology, University Hospital Frankfurt and Goethe University, Frankfurt, Germany; ^4^Department of Neurosurgery, University Hospital Frankfurt and Goethe University, Frankfurt, Germany

**Keywords:** longitudinal follow-up after epilepsy surgery, cognitive decline, improvement in quality of life, reliable change index, standardized regression-based change norms, minimal clinically important difference

## Abstract

**Objective:**

The establishment of patient-centered measures capable of empirically determining meaningful cognitive change after surgery can significantly improve the medical care of epilepsy patients. Thus, this study aimed to develop reliable change indices (RCIs) and standardized regression-based (SRB) change norms for a comprehensive neuropsychological test battery in the German language.

**Methods:**

Forty-seven consecutive patients with temporal lobe epilepsy underwent neuropsychological assessments, both before and 12 months after surgery. Practice-effect-adjusted RCIs and SRB change norms for each test score were computed. To assess their usefulness, the presented methods were applied to a clinical sample, and binary logistic regression analyses were conducted to model the odds of achieving improvement in quality of life (QOL) after surgery.

**Results:**

The determined RCIs at 90% confidence intervals and the SRB equations for each test score included in the test battery are provided. Cohen’s kappa analyses revealed a moderate mean agreement between the two measures, varying from slight to almost perfect agreement across test scores. Using these measures, a negative association between improvement in QOL and decline in verbal memory functions after surgery was detected (adjusted odds ratio = 0.09, *p* = 0.006).

**Significance:**

To the best of our knowledge, this study is the first to develop RCIs and SRB change norms necessary for the objective determination of neuropsychological change in a comprehensive test battery in the German language, facilitating the individual monitoring of improvement and decline in each patients’ cognitive functioning and psychosocial situations after epilepsy surgery. The application of the described measures revealed a strong negative association between improvement in QOL and decline in verbal memory functions after surgery.

## Introduction

In approximately 30% of epilepsy patients, anti-seizure drugs (ASD) fail to sufficiently control seizures (Sander, 2003). Temporal lobe epilepsy (TLE) represents the most common subgroup of drug resistant focal epilepsies (Engel, 2001). As demonstrated by numerous studies (Wiebe et al., 2001; Engel et al., 2012; Engel, 2018; Mohan et al., 2018), for selected drug-resistant patients, epilepsy surgery is considered to be a safe, evidence-based, and effective treatment option that is superior to continued medical therapy. Although seizure control remains the primary aim and is the most examined outcome of epilepsy surgery, the investigation of treatment effects on the patients’ cognitive functioning and psychosocial situations has gained increasing importance in the field. Through the repeated administration of standardized psychometric tests and self-report questionnaires, clinical neuropsychology aims (1) to quantify the presurgical functionality of affected brain structures to facilitate individualized predictions of possible cognitive risks (Elger et al., 2004; Helmstaedter, 2004, 2008) and (2) to evaluate postsurgical cognitive and behavioral change and its impact on the patients’ quality of life (QOL) (Baxendale, 2008; Sherman et al., 2011; Ives-Deliperi and Butler, 2017).

Traditionally, unstandardized difference scores and arbitrary cut-off values were applied to determine cognitive and behavioral change (either across time or in response to intervention). However, the assessment of neuropsychological change has been associated with several psychometric difficulties, as change in test results can be attributed to multiple factors that are unrelated to the intervention investigated (e.g., low retest reliability, or measurement errors). Therefore, empirical methods are required to determine whether change in repeated neuropsychological test results represent true change, which can be considered statistically meaningful, or change caused by random fluctuations in measurements (Hermann et al., 1996; Chelune and Franklin, 2003; Perdices, 2005).

1. The reliable change index (RCI), which was originally introduced by Jacobson and Truax (1991), is the most popular empirical technique for the assessment of neuropsychological change. RCIs are computed as ratios, in which the difference between retest- and baseline scores is divided by an error term (standard error of measurement of the difference). Because the calculation of the error term has been continuously debated among researchers, numerous modified RCI approaches exist in the literature, and the choice of RCI approach should be directed by practical and theoretical considerations (Perdices, 2005).
2. Standardized regression-based (SRB) change norms were initially developed by McSweeny et al. (1993) and are based on multiple regression modeling. During this statistical procedure, baseline scores, together with moderating demographic and clinical variables, are used to derive regression equations to predict retest scores. These predictions are compared to the observed retest scores, and differences are *z*-transformed.
3. The minimal clinically important difference (MCID) describes the amount of change, i.e., the smallest difference between retest- and baseline scores in self-report questionnaires, which a patient considers to be important (McGlothlin and Lewis, 2014). Thus, MCID is a patient-centered approach that not only incorporates the magnitude of change but also its value for the patient. Previous studies have provided thresholds for MCID in commonly used (epilepsy-specific) questionnaires (Wiebe et al., 2002; Button et al., 2015).

Despite their superior informative value, these patient-centered empirical methods have been incorporated into neuropsychological research and clinical routine rather slowly. As reviewed by Sherman et al. (2011), many clinicians continue to apply unstandardized measures, and studies often focus on group-level analyses, during which the results of patients who experience improvement, decline, and no change between assessments are aggregated, which can mask individual change. The slow implementation of empirical measures may be due to availability problems. Although publications examining empirical measures for a wide range of more popular, mostly psychometric tests in the English language exist, data for specific, less widely used tests in languages other than English is often less readily available (Zahra and Hedge, 2010).

The primary objective of this study was to address this issue by developing empirical measures for a comprehensive neuropsychological test battery in the *German language*. To the best of our knowledge, this study is the first to develop both RCIs and SRB change norms for the assessment of meaningful change for *each test score* included in the standard test battery, as recommended by the German ILAE Chapter and the Austrian, German and Swiss Working Group on Presurgical Epilepsy Diagnosis and Epilepsy Surgery (Brückner, 2012; Rosenow et al., 2016).

A secondary objective was to illustrate how the application of the presented measures (RCIs, SRB change norms, and MCID thresholds) can facilitate the objective determination of cognitive and behavioral change in individual patients, by following a clinical sample of TLE patients over 12 months after epilepsy surgery. Improvement and decline in the patients’ cognitive functioning and psychosocial situations were monitored individually, and factors that affected improvement in QOL after surgery were assessed.

## Materials and Methods

### Patients

This study used longitudinal data of a consecutive clinical sample of 50 TLE patients, who underwent epilepsy surgery at the Epilepsy Center Frankfurt Rhine-Main. Patients without formal education (*n* = 2) or with diagnosed psychiatric comorbidities (*n* = 1) were excluded (6.0%), resulting in a final sample of 47 patients (70.2% women; mean age: 32.8 years, *SD* = 12.0). The study was approved by the Ethics Committee of the University of Frankfurt Medical Faculty. The informed consent was waived by the ethics committee because of the retrospective nature of the analysis. Epilepsy syndrome diagnoses were obtained during video-EGG-monitoring, and the classifications of epilepsies and etiologies were based on the latest definitions proposed by the ILAE (Fisher et al., 2017; Scheffer et al., 2017) and the four-dimensional epilepsy classification (Lüders et al., 2019a,b; Rosenow et al., 2020). The neuropsychological assessments followed the standards established by the German ILAE Chapter and the Austrian, German, and Swiss Working Group on Presurgical Epilepsy Diagnosis and Epilepsy Surgery (Brückner, 2012; Rosenow et al., 2016). A standard neuropsychological test battery and two self-report questionnaires were performed, both before and 12 months after epilepsy surgery. Hemispheric language lateralization was assessed by functional transcranial Doppler sonography as described previously (Conradi et al., 2019) (46.8%), functional MRI (38.3%) or the Wada test (14.9%). All surgeries were conducted within the temporal lobe, and 48.9% of surgeries were conducted within the language-dominant hemisphere (dominant TL surgery), whereas 51.1% were conducted within the non-dominant hemisphere (non-dominant TL surgery). Sixteen patients (34.0%) underwent classical two-third temporal lobectomies, 13 patients (27.7%) received amygdalohippocampectomies including the temporal pole, 2 patients (4.3%) received subtemporal selective amygdalohippocampectomies, and 16 patients (34.0%) received extended lesionectomies. Seizure outcome was classified using the Engel Epilepsy Surgery Outcome Scale (Engel, 1993). The socio-demographic and clinical characteristics of the sample are summarized in Table 1.

**TABLE 1:** Socio-demographic and clinical characteristics of the full sample (*n* = 47) and the two subgroups of patients with dominant TL surgery (*n* = 23) and patients with non-dominant TL surgery (*n* = 24).

	All patients	Dominant TL surgery	Non-dominant TL surgery	
	(*n* = 47)	(*n* = 23)	(*n* = 24)	
	**Number (%)**	**Number (%)**	**Number (%)**	***p*-value^c^**

**Gender**				
Women	33 (70.2%)	17 (73.9%)	16 (66.7%)	0.587
Men	14 (29.8%)	6 (26.1%)	8 (33.3%)	
**Handedness**				
Consistently right-handed (EHI ≥ 50)	42 (89.4%)	22 (95.7%)	20 (83.3%)	
Consistently left-handed (EHI ≤ −50)	3 (6.3%)	0 (0.0%)	3 (12.5%)	0.215
Ambidextrous	2 (4.3%)	1 (4.3%)	1 (4.2%)	
**Education**				
≤ 9 years (German Hauptschulabschluss)	9 (19.2%)	3 (13.0%)	6 (25.0%)	
10–12 years (German Realschulabschluss)	22 (46.8%)	12 (52.2%)	10 (41.7%)	0.560
>12 years (German Abitur)	16 (34.0%)	8 (34.8%)	8 (33.3%)	
**Etiology^a^**				
Hippocampal sclerosis	15 (31.8%)	6 (26.2%)	9 (37.5%)	
Arteriovenous malformation	6 (12.8%)	2 (8.7%)	4 (16.7%)	
Long-term epilepsy-associated tumor^b^	14 (29.8%)	9 (39.1%)	5 (20.8%)	0.665
Focal cortical dysplasia	6 (12.8%)	3 (13.0%)	3 (12.5%)	
Unknown	6 (12.8%)	3 (13.0%)	3 (12.5%)	
**Overall seizure frequency before surgery^a^**				
≥ 1 seizures per day	10 (21.3%)	7 (30.4%)	3 (12.5%)	
1–6 seizures per week	20 (42.6%)	6 (26.2%)	14 (58.4%)	
1–3 seizures per month	14 (29.7%)	9 (39.1%)	5 (20.8%)	0.100
1–11 seizures per year	3 (6.4%)	1 (4.3%)	2 (8.3%)	
**Focal to bilateral tonic-clonic seizures^a^**				
Never occurred before surgery	14 (29.8%)	8 (34.8%)	6 (25.0%)	0.464
Occurred (at least once) before surgery	33 (70.2%)	15 (65.2%)	18 (75.0%)	
**Surgical procedure**				
Classical two-third temporal lobectomy	16 (34.0%)	7 (30.4%)	9 (37.5%)	
Amygdalohippocampectomy incl. temporal pole	13 (27.7%)	5 (21.7%)	8 (33.3%)	0.589
Selective amygdalohippocampectomy	2 (4.3%)	1 (4.3%)	1 (4.2%)	
Extended lesionectomy	16 (34.0%)	10 (43.6%)	6 (25.0%)	
**Seizure outcome after surgery**				
Engel class I (free of disabling seizures)	33 (70.2%)	18 (78.3%)	15 (62.5%)	
Engel class II (rare disabling seizures)	8 (17.0%)	2 (8.7%)	6 (25.0%)	
Engel class III (worthwhile improvement)	2 (4.3%)	1 (4.3%)	1 (4.2%)	0.522
Engel class IV (no worthwhile improvement)	4 (8.5%)	2 (8.7%)	2 (8.3%)	

	**Mean (*SD*)**	**Mean (*SD*)**	**Mean (*SD*)**	***p*-value^d^**

Age (years)	32.8 (12.0)	31.0 (12.6)	34.6 (11.3)	0.303
Age at onset of epilepsy (years)	19.4 (10.9)	18.4 (11.0)	20.3 (11.0)	0.548
Duration of epilepsy (years)	13.4 (12.0)	12.6 (11.4)	14.3 (12.7)	0.634
**Intelligence**				
Socio-demographic prediction model (IQ)	100.1 (9.0)	100.2 (9.3)	100.0 (8.9)	0.947
MWT-B (Verbal IQ)	102.6 (9.7)	99.1 (9.9)	106.0 (8.4)	0.014*

*SD*, standard deviation. ^*a*^Syndrome diagnoses were obtained during video-EGG-monitoring. ^*b*^Including 9 grade I gangliogliomas, 4 dysembryoplastic neuroepithelial tumors and 1 grade I astrocytoma. ^*c*^Chi-Square tests and ^*d*^independent-samples *t*-tests, *p < 0.05.

### Neuropsychological Assessments

All neuropsychological assessments were performed in a standardized fashion, by trained neuropsychologists, and lasted approximately 3 h each. Patients were verified as not currently being treated with topiramate, receiving no acute treatments with benzodiazepines, and not having seizures or status epilepticus within the 24 h immediately before or during the assessments.

The neuropsychological test battery recommended by the German ILAE Chapter and the Austrian, German and Swiss Working Group on Presurgical Epilepsy Diagnosis and Epilepsy Surgery (Brückner, 2012; Rosenow et al., 2016) was administered at the pre- and postsurgical assessments, consisting of the following standardized psychometric tests, as described elsewhere (Conradi et al., 2020): (1) To evaluate *attentional functions*, the subtest Divided Attention of the computerized “Testbatterie zur Aufmerksamkeitsprüfung” (TAP) (Zimmermann and Fimm, 2007) was applied. (2) For the assessment of *verbal learning and memory functions*, the “Verbaler Lern- und Merkfähigkeitstest” (VLMT) (Helmstaedter et al., 2001) was used. (3) For the evaluation of *non-verbal learning and memory functions*, the “Diagnosticum für Cerebralschädigung II” (DCS-II) (Weidlich et al., 2011) was performed. (4) Additionally, the “Rey-Osterrieth Complex Figure Test” (ROCFT) (Rey, 1941; Osterrieth, 1944) was used. (5) To measure *short-term memory and working memory*, the subtests Digit Span and Visual Memory Span of the “Wechsler Memory Scale-Revised” (WMS-R) (Härting et al., 2000) were performed. (6) *Visuospatial functioning* was measured by the completeness of the copy of the complex figure from the ROCFT. (7) Moreover, the subtests Silhouettes and Position Discrimination of the “Visual Object and Space Perception Battery” (VOSP) (Warrington and James, 1992) were used. (8) *Language functions* were assessed by the phonemic and semantic verbal fluency subtests of the “Regensburger Wortflüssigkeitstest” (RWT) (Aschenbrenner et al., 2001). (10) To assess an aspect of *executive functioning*, the Flexibility subtest of the TAP was used.

During both assessments, self-reported *symptoms of depression* were evaluated using the 21-item “Beck Depression Inventory-II” (BDI-II) (Beck et al., 1996), with higher scores indicating greater severity of symptoms (0–13: minimal depression; 14–19: mild depression; 20–28: moderate depression; and 29–63: severe depression). The 31-item “Quality of Life in Epilepsy Inventory-31” (QOLIE-31) (Devinsky et al., 1995) was used during both assessments, to measure the self-reported *quality of life* on seven subscales (seizure worry, overall QOL, emotional well-being, energy and fatigue, cognitive functioning, medication effects, and social functioning), which were combined for a total score, with standardized scores ranging from 0 to 100 and higher scores demonstrating better QOL.

Additionally, during the presurgical assessments, intelligence was estimated using a prediction formula based on socio-demographic characteristics (Jahn et al., 2013), verbal IQ was measured using a multiple-choice vocabulary test (“Mehrfachwahl-Wortschatz-Intelligenztest”, MWT-B) (Lehrl, 1999), and handedness was determined by the “Edinburgh Handedness Inventory” (EHI) (Oldfield, 1971).

### Statistical Analyses

Test results obtained during the presurgical assessments (t_1_) are referred to as “baseline scores,” whereas test results from the postsurgical assessments (t_2_) are referred to as “retest scores.” The median interval between t_1_ and epilepsy surgery was 6 months (*SD* = 14.47), between epilepsy surgery and t_2_ was 12 months (*SD* = 1.01), and between t_1_ and t_2_ was 18 months (*SD* = 14.05).

Analyses of the socio-demographic and clinical characteristics of the full sample and the two subgroups (dominant vs. non-dominant TL surgery) were conducted using independent-samples *t*-tests for numerical data and chi-square tests for categorical data. Paired-samples *t*-tests were applied to examine differences between retest and baseline scores on group-level. Analyses were performed in IBM SPSS Statistics 22 (IBM Corporation, Armonk, NY, United States) and Microsoft Excel 2016 (Microsoft, Seattle, WA, United States).

#### Development of the Presented Measures

To not only allow determining the degree of expected change in repeated neuropsychological test results associated with random fluctuations in measurements (e.g., practice with test materials), but to also take the influence of disease-related factors (e.g., continued medical therapy, or brain surgery in general) into account, the computation of RCIs and SRB change norms was based on data obtained from a surgical cohort of epilepsy patients. However, to preclude expected *material-specific* cognitive change after brain surgery [dominant TL surgery often associated with decline in verbal learning and memory functions, and non-dominant TL surgery often associated with decline in non-verbal learning and memory functions (Sherman et al., 2011)], the calculations for VLMT test scores were only based on data from the subgroup of patients with non-dominant TL surgery. Accordingly, calculations for DCS-II and ROCFT test scores were only based on data from the subgroup of patients with dominant TL surgery. All other calculations were based on the full sample.

Cohen’s kappa analyses were performed to assess the agreement between the two computed measures (RCIs and SRB change norms) in each neuropsychological test score.

##### Reliable change indices

Practice-effect (PE)-adjusted RCIs for each test score included in the neuropsychological test battery were computed according to the approach described by Chelune et al. (1993). First, retest reliability coefficients (*r*_*t*__1__t__2_) for each test score were obtained. Then, the standard error of measurement of the difference (*SE*_*diff*_) for each test score was computed as follows: *SE*_*diff*_ = [*SD*_*t*__1_ × 2 (1 – *r*_*t*__1__t__2_)^1/2^] – PE. Next, 90% confidence intervals were generated for the resulting *SE*_*diff*_ scores by multiplying them by ± 1.64. To adjust for practice effects, the mean change between t_2_ and t_1_ for each particular test score was added to the upper and lower limits of the confidence interval.

##### Standardized regression-based change norms

Utilizing the methods described by McSweeny et al. (1993), multiple linear regression analyses were conducted to predict the retest score for each neuropsychological test score using the baseline score combined with potential moderating demographic and clinical variables (intelligence, age at t_1_, age at onset of epilepsy, duration of epilepsy, and the interval between t_1_ and t_2_) confirmed in previous studies (Hermann et al., 1996; Busch et al., 2015). Collinearity statistics were computed to preclude concerns regarding multicollinearity between the predictor variables, and a stepwise procedure (0.05 = threshold for variable entry; 0.10 = threshold for variable removal) was used.

#### Longitudinal Follow-Up of TLE Patients

The computed RCIs and SRB change norms were applied to determine meaningful change for each patient in each test score included in the neuropsychological test battery. The MCID in each scale of the self-report questionnaires was assessed for each patient using the thresholds provided in the literature (Wiebe et al., 2002; Button et al., 2015). The proportions of patients who achieved meaningful change in each test score and scale were compared between the two subgroups (dominant vs. non-dominant TL surgery) using chi-square tests.

To investigate the patients’ clinical characteristics and neuropsychological factors that influenced improvement in QOL after surgery, univariate binary logistic regression analyses were computed. Crude odds ratios and respective 95% confidence intervals were used to measure the magnitude of associations between improvement in QOL and the predictor variables. Prior to conducting multiple binary logistic regression analyses, the suitability of the data characteristics for this operation was confirmed. A forward stepwise procedure (Wald, 0.05 = threshold for variable entry; 0.10 = threshold for variable removal) was used, and an adjusted odds ratio and the respective 95% confidence interval for each included predictor variable were reported. The overall percentage accuracy in classification (PAC), specificity, sensitivity, and Nagelkerke *R*^2^ were computed to evaluate the quality of the resulting models.

## Results

Twelve months after surgery, 33 patients were free of disabling seizures (70.2%, Engel class I), among which 29 patients were completely seizure-free since surgery (Engel class IA). Eight patients had rare disabling seizures (17.0%, Engel class II), two patients achieved a worthwhile improvement (4.3%, Engel class III), and four patients experienced no worthwhile improvement in seizure control (8.5%, Engel class IV) after surgery. No significant differences were identified between the two subgroups (dominant vs. non-dominant TL surgery) with regard to socio-demographic and clinical characteristics, except for verbal IQ, which was lower in patients with an epileptogenic focus in the language-dominant hemisphere (Table 1).

A summary of the mean baseline- and retest raw scores, the mean change scores and results of the group-level analyses, and the retest reliability coefficients for each test score included in the neuropsychological test battery and each scale of the self-report questionnaires are presented in Table 2.

**TABLE 2:** Mean raw scores, standard deviation (*SD*) and mean change between t_2_ and t_1_ (compared by paired samples *t*-tests) together with retest reliability coefficients for each test score and scale.

Test score	t_1_ Mean (*SD*)	t_2_ Mean (*SD*)	Mean change	Retest reliability
***Attention***				
**TAP Divided Attention**				
Auditory reaction times	586.66 (80.59)	589.23 (92.62)	2.57	0.45
Visual reaction times	794.96 (78.56)	808.17 (211.37)	13.21	0.43
Auditory omissions	0.64 (0.74)	0.30 (0.66)	−0.34*	0.23
Visual omissions	0.94 (1.13)	1.19 (1.58)	0.26	0.49
Total omissions	1.51 (1.43)	1.47 (1.92)	–0.04	0.53
Mistakes	2.85 (6.27)	3.06 (4.16)	0.21	0.18
***Learning and memory***				
**VLMT^a^**				
First repetition, LW	7.58 (1.93)	7.25 (2.17)	–0.33	0.34
Fifth repetition, LW	12.71 (2.01)	12.79 (2.13)	0.08	0.46
All repetitions, LW	53.92 (9.49)	54.71 (10.03)	0.79	0.46
Distraction list, LW	6.63 (1.58)	6.42 (2.28)	–0.21	0.26
Recall a. distraction, FW	1.88 (1.51)	2.04 (2.14)	0.17	0.41
Delayed recall	10.17 (3.47)	10.71 (3.54)	0.54	0.46
Delayed recall, FW	2.67 (2.01)	2.13 (2.25)	–0.54	0.28
Recognition	14.21 (0.93)	13.88 (2.07)	–0.33	0.50
Recognition, mistakes	1.25 (1.80)	1.63 (3.06)	0.38	0.75
Interferences	0.29 (0.69)	0.13 (0.45)	–0.17	0.32
False positives	0.79 (1.53)	1.08 (2.08)	0.29	0.21
Repetitions	4.54 (3.24)	5.54 (4.98)	1.00	0.25
**DCS-II^b^**				
First repetition, LF	2.83 (1.64)	3.78 (1.68)	0.96	0.14
All repetitions, LF	24.26 (16.59)	20.87 (13.22)	–3.39	0.50
**ROCFT**^b^				
Delayed recall	14.37 (5.94)	18.65 (6.00)	4.28**	0.59
**WMS-R**				
Digit span, forwards	6.36 (1.89)	7.17 (2.06)	0.81**	0.56
Digit span, backwards	5.98 (1.89)	6.30 (2.06)	0.33	0.44
Visual memory span, forwards	8.47 (2.01)	8.32 (2.18)	–0.15	0.66
Visual memory span, backwards	7.72 (2.21)	7.38 (2.17)	–0.34	0.63
***Visuospatial functioning***				
**ROCFT**				
Copy	34.28 (2.60)	34.00 (3.68)	–0.28	0.78
**VOSP**				
Silhouettes	20.21 (3.67)	22.45 (3.80)	2.23***	0.58
Position discrimination	19.81 (0.45)	19.51 (1.33)	–0.30	0.02
***Language***				
**RWT verbal fluency**				
Phonemic, one letter	14.34 (4.35)	13.45 (4.07)	–0.89	0.33
Phonemic, two letters	12.43 (4.46)	12.45 (3.74)	0.02	0.34
Semantic, one category	21.89 (7.43)	18.98 (6.62)	−2.91**	0.67
Semantic, two categories	14.98 (3.67)	13.66 (3.41)	−1.32*	0.46
***Executive functioning***				
**TAP Flexibility**				
Reaction times	796.45 (226.48)	694.30 (188.91)	−102.15**	0.51
Mistakes	2.89 (3.29)	2.28 (2.37)	–0.62	0.51
***Symptoms of depression***				
**BDI-II**	11.64 (8.79)	6.68 (6.20)	−4.96***	0.46
***Quality of life***				
**QOLIE-31**				
Seizure worry	46.17 (22.26)	79.43 (20.17)	13.00***	0.14
Overall quality of life	62.43 (16.69)	71.21 (16.67)	4.94**	0.25
Emotional well-being	60.47 (16.20)	68.23 (16.87)	3.94**	0.44
Energy and fatigue	47.60 (13.30)	54.87 (17.16)	3.36**	0.54
Cognitive functioning	54.60 (20.34)	61.40 (19.04)	3.00*	0.47
Medication effects	51.38 (22.05)	59.64 (22.48)	2.74*	0.23
Social functioning	50.64 (12.68)	54.68 (9.28)	1.51	0.22
Total score	54.40 (11.66)	63.06 (12.34)	8.66***	0.47

LW, learned words; FW, forgotten words; LF, learned figures. Mean raw scores, *SD*, mean change and paired samples *t*-tests only based on data from the subgroup of ^*a*^patients with non-dominant TL surgery or ^*b*^patients with dominant TL surgery. *p < 0.05, **p < 0.01, ***p < 0.001.

### Reliable Change Indices

The PE-adjusted RCIs at 90% confidence intervals for each neuropsychological test score are provided in Table 3.

**TABLE 3:** Practice-effect (PE)-adjusted reliable change indices (RCIs) and respective 90% confidence intervals (CI) for each neuropsychological test score.

Test score	Correction for PE	Adjusted RCIs (90% CI)
***Attention***		
**TAP Divided Attention**		
Auditory reaction times	3	−140.97 to 135.82
Visual reaction times	13	−150.39 to 123.97
Auditory omissions	0	−1.16 to 1.84
Visual omissions	0	−2.12 to 1.61
Total omissions	0	−2.23 to 2.31
Mistakes	0	−13.37 to 12.94
***Learning and memory***		
**VLMT^a^**		
First repetition, LW	0	−3.35 to 4.02
Fifth repetition, LW	0	−3.49 to 3.32
All repetitions, LW	1	−17.73 to 16.15
Distraction list, LW	0	−3.94 to 4.36
Recall a. distraction, FW	0	−3.45 to 3.12
Delayed recall	1	−6.53 to 5.45
Delayed recall, FW	−1	−3.85 to 4.93
Recognition	0	−1.70 to 2.36
Recognition, mistakes	0	−3.03 to 2.28
Interferences	0	−1.12 to 1.45
False positives	0	−4.14 to 3.56
Repetitions	1	−8.37 to 6.37
**DCS-II**^b^		
First repetition, LF	1	−4.40 to 2.49
All repetitions, LF	−3	−21.71 to 28.50
**ROCFT^b^**		
Delayed recall	4	−12.24 to 3.68
**WMS-R**		
Digit span, forwards	1	−3.72 to 2.11
Digit span, backwards	0	−3.61 to 2.96
Visual memory span, forwards	0	−2.59 to 2.88
Visual memory span, backwards	0	−2.77 to 3.45
***Visuospatial functioning***		
**ROCFT**		
Copy	0	−2.54 to 3.09
**VOSP**		
Silhouettes	2	−7.77 to 3.30
Position discrimination	0	−0.75 to 1.35
***Language***		
**RWT verbal fluency**		
Phonemic, one letter	−1	−7.35 to 9.14
Phonemic, two letters	0	−8.39 to 8.35
Semantic, one category	−3	−6.99 to 12.82
Semantic, two categories	−1	−4.95 to 7.59
***Executive functioning***		
**TAP Flexibility**		
Reaction times	−102	263.98 to 468.28
Mistakes	−1	−4.75 to 5.98

*LW, learned words; FW, forgotten words; LF, learned figures. ^*a*^Practice-effect (PE) only obtained from the subgroup of patients with non-dominant TL surgery. ^*b*^Practice-effect (PE) only obtained from the subgroup of patients with dominant TL surgery.*

#### Application

Individual change scores (i.e., differences between retest- and baseline scores) for each test score for each patient have to be computed. Change scores that fall within the RCI interval represent change expected to occur by chance in 90% of cases. Change scores that exceed the RCI interval indicate meaningful change.

#### Example

A patient achieving a 145-ms improvement in auditory reaction times between t_2_ and t_1_ (which exceeds the lower limit of the RCI interval, as depicted in Table 3) would show a clinically meaningful change. In contrast, an improvement of 145 ms in visual reaction times (which falls within the RCI interval) would be considered insignificant.

### Standardized Regression-Based Change Norms

The SRB equations for each neuropsychological test score, derived from multiple linear regression analyses, are presented in Table 4. Where applicable, one equation with and one without moderating demographic and clinical variables (MV) is provided. The respective baseline score was a significant predictor of the retest score in 67.7% of equations.

**TABLE 4:** SRB equations for each neuropsychological test score, with and without moderating demographic and clinical variables (MV) where applicable.

Test score	*R*	*SE*_est_	B	B_IQ_	B_Age_	B_Onset_	B_Duration_	B_Interval_
***Attention***								
**TAP Divided Attention**								
Auditory reaction times	0.99	91.27	1.00					
Auditory reaction times (MV)	0.99	80.90	0.80		3.53			
Visual reaction times	0.97	190.86	1.02					
Visual reaction times (MV)	–	–	–					
Auditory omissions	–	–	–					
Auditory omissions (MV)	0.50	0.62					0.02	
Visual omissions	0.69	1.44	0.93					
Visual omissions (MV)	0.74	1.36	0.67		0.02			
Total omissions	0.73	1.65	0.85					
Total omissions (MV)	–	–	–					
Mistakes	–	–	–					
Mistakes (MV)	0.73	3.57						0.14
***Learning and memory***								
**VLMT**^a^								
First repetition, LW	–	–	–					
First repetition, LW (MV)	0.96	2.02		0.07				
Fifth repetition, LW	0.99	1.47	1.00					
Fifth repetition, LW (MV)	–	–	–					
All repetitions, LW	0.99	8.04	1.01					
All repetitions, LW (MV)	0.99	7.41	0.60	0.22				
Distraction list, LW	–	–	–					
Distraction list, LW (MV)	0.95	2.16		0.06				
Recall after distraction, FW	–	–	–					
Recall after distraction, FW (MV)	0.74	2.01				0.09		
Delayed recall	0.96	3.04	1.01					
Delayed recall (MV)	0.97	2.75	0.59	0.05				
Delayed recall, FW	–	–	–					
Delayed recall, FW (MV)	0.72	2.16				0.10		
Recognition	0.99	1.74	0.98					
Recognition (MV)	0.99	1.50	1.10			−0.08		
Recognition, mistakes	0.82	2.02	1.29					
Recognition, mistakes (MV)	–	–	–					
Interferences	–	–	–					
Interferences (MV)	0.41	0.43					0.01	
False positives	–	–	–					
False positives (MV)	0.46	2.09		0.01				
Repetitions	0.76	4.94	1.01					
Repetitions (MV)	–	–	–					
**DCS-II**^b^								
First repetition, LF	–	–	–					
First repetition, LF (MV)	0.92	1.67		0.04				
All repetitions, LF	–	–	–					
All repetitions, LF (MV)	0.85	13.29		0.21				
**ROCFT**^b^								
Delayed recall	0.95	6.01	1.20					
Delayed recall (MV)	0.97	5.06	0.64	0.09				
**WMS-R**								
Digit span, f.	0.97	1.95	1.09					
Digit span, f. (MV)	0.98	1.67	0.63	0.03			0.04	
Digit span, b.	–	–	–					
Digit span, b. (MV)	0.96	1.90	0.49	0.03				
Visual memory span, f.	0.98	1.73	0.97					
Visual memory span, f. (MV)	0.98	1.61	0.67	0.03				
Visual memory span, b.	0.97	1.83	0.93					
Visual memory span, b. (MV)	0.98	1.59	0.56	0.04			−0.05	
***Visuospatial functioning***								
**ROCFT**								
Copy	0.99	2.31	0.99					
Copy (MV)	–	–	–					
**VOSP**								
Silhouettes	0.99	3.61	1.09					
Silhouettes (MV)	0.99	3.03	0.63	0.08				0.09
Position discrimination	0.99	1.41	0.98					
Position discrimination (MV)	–	–	–					
***Language***								
**RWT verbal fluency**								
Phonemic, one letter	–	–	–					
Phonemic, one letter (MV)	0.97	3.75	0.27	0.10				
Phonemic, two letters	–	–	–					
Phonemic, two letters (MV)	0.96	3.61		0.12				
Semantic, one category	0.97	5.27	0.84					
Semantic, one category (MV)	0.98	4.46	0.60	0.11	−0.15			
Semantic, two categories	–	–	–					
Semantic, two categories (MV)	0.98	3.03	0.42	0.07				
***Executive functioning***								
**TAP Flexibility**								
Reaction times	0.97	188.62	0.84					
Reaction times (MV)	0.98	161.20	0.43	2.19	4.07			
Mistakes	0.73	2.24	0.55					
Mistakes (MV)	0.78	2.08	0.38	0.01				

**R* = reliability coefficient, *SE*_*est*_ = standard error of the estimate, *B* = unstandardized beta for respective baseline score, *B*_*IQ*_ = unstandardized beta for intelligence as assessed by a socio-demographic prediction model, *B*_*Age*_ = unstandardized beta for age at t_1_, *B*_*Onset*_ = unstandardized beta for age at onset of epilepsy, *B*_*Duration*_ = unstandardized beta for duration of epilepsy, *B*_*Interval*_ = unstandardized beta for interval between t_1_ and t_2_. LW, learned words; FW, forgotten words; LF, learned figures. ^*a*^Calculations only based on data from the subgroup of patients with non-dominant TL surgery. ^*b*^Calculations only based on data from the subgroup of patients with dominant TL surgery.*

#### Application

By using the SRB equations, the retest scores for each patient in each test score can be predicted. Each difference between the predicted and the observed retest score can then be transformed into a standardized *z*-score (SRB change score) by dividing the difference by the respective standard error of the estimate (*SE*_*est*_). SRB change scores that exceed the 90% confidence interval (*z* = ± 1.64) indicate meaningful change.

#### Example

A 49-year-old patient achieves a baseline auditory reaction time of 495 ms and a retest auditory reaction time of 431 ms. By using the respective SRB equation from Table 4, the patients’ retest score can be predicted as follows: predicted retest score = (*B* × baseline score) + (*B*_*A*__*ge*_ × age at t_1_) = (0.80 × 495) + (3.53 × 49) = 568.97. The difference between the predicted retest score (568.97) and the observed retest score (431) can then be *z*-transformed as follows: SRB change score = (observed retest score – predicted retest score)/*SE*_*est*_ = (431 –568.97)/80.90 = −1.71. Because the resulting SRB change score exceeds the 90% confidence interval at the lower limit, this 64-ms improvement between t_2_ and t_1_ can be interpreted as a meaningful change.

### Comparison Between RCIs and SRB Change Norms

Cohen’s kappa analyses revealed a moderate mean agreement (Landis and Koch, 1977) between RCIs and SRB change norms across test scores (mean κ = 0.44, *SD* = 0.26). The coefficients ranged between slight agreement (κ = 0.05, *p* = 0.511) for the subtest Digit Span forwards of the WMS-R, and almost perfect agreement (κ = 0.91, *p* = 0.000) for repetitions in the VLMT. SRB change norms were more conservative (i.e., indicating no meaningful change more frequently) in 72.3% of cases. Therefore, only those results based on SRB change norms are reported in the following.

### Application of the Presented Measures

According to MCID thresholds, after surgery, 34 patients (72.4%) experienced meaningful improvement in symptoms of depression, and 20 patients (42.5%) achieved meaningful improvement in QOL. After surgery, 39 patients (83.0%) reported reduced seizure worry, 23 patients (48.9%) reported improved overall QOL, 20 patients (42.5%) reported improved emotional well-being, 17 patients (36.2%) reported improved energy, 14 patients (29.8%) reported improved cognitive functioning, 18 patients (38.3%) reported reduced medication side effects, and 14 patients (29.8%) reported improved social functioning. A decline in mood was less frequent than an improvement or no change in every scale of the self-report questionnaires.

The proportions of patients who experienced decline, improvement, or no meaningful change in each neuropsychological test score (according to SRB change norms) and each scale of the self-report questionnaires (according to MCID thresholds) were computed separately for the two subgroups and are presented in Table 5. Chi-Square tests revealed that a significantly higher proportion of patients who underwent dominant compared to non-dominant TL surgery experienced decline in verbal learning functions (VLMT, fifth repetition, learned words: 47.8 vs. 4.2%, *p* = 0.001; all repetitions, learned words: 39.1 vs. 4.2%, *p* = 0.003) and verbal memory functions (VLMT, recall after distraction, forgotten words: 17.4 vs. 0.0%, *p* = 0.033; delayed recall: 39.1 vs. 8.3%, *p* = 0.013; recognition, mistakes: 47.8 vs. 4.2%, *p* = 0.001; interferences: 34.8 vs. 8.3%, *p* = 0.027) after surgery. The proportion of patients with non-dominant TL surgery that achieved improvement in emotional well-being (62.5 vs. 21.7%, *p* = 0.005) and cognitive functioning (45.8 vs. 13.0%, *p* = 0.014) after surgery was significantly higher compared to patients with dominant TL surgery.

**TABLE 5:** Proportion of patients achieving decline, improvement or no meaningful change in each neuropsychological test score (according to SRB change norms) and each scale of the self-report questionnaires (according to MCID thresholds), computed separately for the two subgroups (compared by chi-square tests).

	Dominant TL surgery (*n* = 23)			Non-dominant TL surgery (*n* = 24)		
	Decline	No change	Improvement	Decline	No change	Improvement
Test score^a^	*n* (%)	*n* (%)	*n* (%)	*n* (%)	*n* (%)	*n* (%)
***Attention***						
**TAP Divided Attention**						
Auditory reaction times	0 (0.0)	22 (95.7)	1 (4.3)	3 (12.5)	20 (83.3)	1 (4.2)
Visual reaction times	0 (0.0)	23 (100)	0 (0.0)	1 (4.2)	23 (95.8)	0 (0.0)
Auditory omissions	1 (4.3)	22 (95.7)	0 (0.0)	2 (8.3)	22 (91.7)	0 (0.0)
Visual omissions	0 (0.0)	23 (100)	0 (0.0)	2 (8.3)	22 (91.7)	0 (0.0)
Total omissions	1 (4.3)	22 (95.7)	0 (0.0)	2 (8.3)	22 (91.7)	0 (0.0)
Mistakes	2 (8.7)	21 (91.3)	0 (0.0)	2 (8.3)	22 (91.7)	0 (0.0)
***Learning and memory***						
**VLMT**						
First repetition, LW	2 (8.7)	20 (87.0)	1 (4.3)	1 (4.2)	22 (91.7)	1 (4.2)
Fifth repetition, LW**	11 (47.8)	12 (52.2)	0 (0.0)	1 (4.2)	22 (91.7)	1 (4.2)
All repetitions, LW**	9 (39.1)	13 (56.5)	1 (4.3)	1 (4.2)	22 (91.7)	1 (4.2)
Distraction list, LW	0 (0.0)	21 (91.3)	2 (8.7)	1 (4.2)	22 (91.7)	1 (4.2)
Recall a. distraction, FW*	4 (17.4)	19 (82.6)	0 (0.0)	0 (0.0)	24 (100)	0 (0.0)
Delayed recall*	9 (39.1)	13 (56.5)	1 (4.3)	2 (8.3)	22 (91.7)	0 (0.0)
Delayed recall, FW	5 (21.7)	18 (78.3)	0 (0.0)	1 (4.2)	23 (95.8)	0 (0.0)
Recognition	6 (26.1)	16 (69.9)	1 (4.3)	1 (4.2)	23 (95.8)	0 (0.0)
Recognition, mistakes**	11 (47.8)	12 (52.2)	0 (0.0)	1 (4.2)	22 (91.7)	1 (4.2)
Interferences*	8 (34.8)	15 (65.2)	0 (0.0)	2 (8.3)	22 (91.7)	0 (0.0)
False positives	4 (17.4)	19 (82.6)	0 (0.0)	2 (8.3)	22 (91.7)	0 (0.0)
Repetitions	1 (4.3)	19 (82.6)	3 (13.0)	2 (8.3)	22 (91.7)	0 (0.0)
**DCS-II**						
First repetition, LF	2 (8.7)	20 (87.0)	1 (4.3)	6 (25.0)	17 (70.8)	1 (4.2)
All repetitions, LF	0 (0.0)	20 (87.0)	3 (13.0)	0 (0.0)	19 (79.2)	5 (20.8)
**ROCFT**						
Delayed recall	0 (0.0)	21 (91.3)	2 (8.7)	3 (12.5)	21 (87.5)	0 (0.0)
**WMS-R**						
Digit span, f.	2 (8.7)	20 (87.0)	1 (4.3)	2 (8.3)	22 (91.7)	0 (0.0)
Digit span, b.	1 (4.3)	20 (87.0)	2 (8.7)	0 (0.0)	24 (100)	0 (0.0)
Visual memory span, f.	1 (4.3)	22 (95.7)	0 (0.0)	3 (12.5)	21 (87.5)	0 (0.0)
Visual memory span, b.	2 (8.7)	21 (91.3)	0 (0.0)	2 (8.3)	22 (91.7)	0 (0.0)
***Visuospatial functioning***						
**ROCFT**						
Copy	1 (4.3)	20 (87.0)	2 (8.7)	1 (4.2)	23 (95.8)	0 (0.0)
**VOSP**						
Silhouettes	3 (13.0)	19 (82.6)	1 (4.3)	0 (0.0)	21 (87.5)	3 (12.5)
Position discrimination	1 (4.3)	22 (95.7)	0 (0.0)	1 (4.2)	22 (91.7)	1 (4.2)
***Language***						
**RWT verbal fluency**						
Phonemic, one letter	1 (4.3)	22 (95.7)	0 (0.0)	4 (16.7)	18 (75.0)	2 (8.3)
Phonemic, two letters	0 (0.0)	22 (95.7)	1 (4.3)	0 (0.0)	21 (87.5)	3 (12.5)
Semantic, one category	1 (4.3)	21 (91.3)	1 (4.3)	3 (12.5)	19 (79.2)	2 (8.3)
Semantic, two categories	2 (8.7)	21 (91.3)	0 (0.0)	0 (0.0)	21 (87.5)	3 (12.5)
***Executive functioning***						
**TAP Flexibility**						
Reaction times	2 (8.7)	21 (91.3)	0 (0.0)	0 (0.0)	24 (100)	0 (0.0)
Mistakes	4 (17.4)	19 (82.6)	0 (0.0)	1 (4.2)	23 (95.8)	0 (0.0)
***Symptoms of depression***						
**BDI-II**	3 (13.0)	4 (17.4)	16 (69.6)	1 (4.2)	5 (20.8)	18 (75.0)
***Quality of life***						
**QOLIE-31**						
Seizure worry	1 (4.3)	2 (8.7)	20 (87.0)	2 (8.3)	3 (12.5)	19 (79.2)
Overall quality of life	4 (17.4)	11 (47.8)	8 (34.8)	3 (12.5)	6 (25.0)	15 (62.5)
Emotional well-being**	2 (8.7)	16 (69.6)	5 (21.7)	1 (4.2)	8 (33.3)	15 (62.5)
Energy and fatigue	2 (8.7)	14 (60.9)	7 (30.4)	3 (12.5)	11 (45.8)	10 (41.7)
Cognitive functioning*	4 (17.4)	16 (69.6)	3 (13.0)	3 (12.5)	10 (41.7)	11 (45.8)
Medication effects	6 (26.1)	10 (43.5)	7 (30.4)	5 (20.8)	8 (33.3)	11 (45.8)
Social functioning	5 (21.7)	12 (52.2)	6 (26.1)	2 (8.3)	14 (58.3)	8 (33.3)
Total score	1 (4.3)	16 (69.6)	6 (26.1)	1 (4.2)	9 (37.5)	14 (58.3)

LW, learned words; FW, forgotten words; LF, learned figures. ^*a*^Chi-Square tests, *p < 0.05, **p < 0.01.

The results of the univariate binary logistic regression analyses used to assess several pre- and postsurgical predictor variables for QOL improvement (QOLIE-31 total score) after surgery are presented in Table 6. Significant associations were observed between improvement in QOL and etiology (long-term epilepsy-associated tumor vs. hippocampal sclerosis, *p* = 0.019), focal to bilateral tonic-clonic seizures (never occurred vs. occurred before surgery, *p* = 0.019), side of surgery (dominant vs. non-dominant TL surgery, *p* = 0.029), verbal memory functions (decline vs. no decline, *p* = 0.018), and baseline QOLIE-31 total score (for every score point higher, *p* = 0.015).

**TABLE 6:** Crude odds ratios (OR) and respective 95% confidence intervals (CI) measuring the association between improvement in quality of life (QOLIE-31 total score) and several pre- and postsurgical predictor variables.

	No improvement in quality of life	Improvement in quality of life		
	(*n* = 27)	(*n* = 20)		
**Predictor variables**	**Number (%)**	**Number (%)**	**Crude OR (95% CI)**	***p*-value**

**Etiology**				
Hippocampal sclerosis	7 (25.9%)	8 (40.0%)	1.0	
Arteriovenous malformation	3 (11.1%)	3 (15.0%)	1.41 (0.25–7.86)	0.694
Long-term epilepsy-associated tumor	12 (44.5%)	2 (10.0%)	0.14 (0.03–0.72)	0.019*
Focal cortical dysplasia	3 (11.1%)	3 (15.0%)	1.41 (0.25–7.86)	0.694
Unknown	2 (7.4%)	4 (20.0%)	3.13 (0.51–19.09)	0.217
**Focal to bilateral tonic-clonic seizures**				
Occurred (at least once) before surgery	15 (55.6%)	18 (90.0%)	1.0	
Never occurred before surgery	12 (44.4%)	2 (10.0%)	0.14 (0.03–0.72)	0.019*
Side of surgery				
Non-dominant TL surgery	10 (37.0%)	14 (70.0%)	1.0	
Dominant TL surgery	17 (63.0%)	6 (30.0%)	0.25 (0.07–0.87)	0.029*
**Surgical procedure**				
Classical two-third temporal lobectomy	7 (25.9%)	9 (45.0%)	1.0	
AHE incl. temporal pole	8 (29.6%)	5 (25.0%)	0.79 (0.21–2.92)	0.726
Selective AHE	0 (0.0%)	2 (10.0%)	–	
Extended lesionectomy	12 (44.4%)	4 (20.0%)	0.31 (0.08–1.19)	0.087
**Seizure outcome after surgery**				
Remaining seizures (Engel class II-IV)	6 (22.2%)	8 (40.0%)	1.0	
Seizure-free (Engel class I)	21 (77.8%)	12 (60.0%)	0.43 (0.12–1.53)	0.192
**Verbal learning functions^a^**				
No decline	15 (55.6%)	14 (70.0%)	1.0	
Decline according to SRB change norms	12 (44.4%)	6 (30.0%)	0.54 (0.16–1.82)	0.316
**Verbal memory functions^b^**				
No decline	12 (44.4%)	16 (80.0%)	1.0	
Decline according to SRB change norms	15 (55.6%)	4 (20.0%)	0.20 (0.05–0.76)	0.018*

	**Mean (*SD*)**	**Mean (*SD*)**	**Crude OR (95% CI)**	***p*-value**

Age at onset of epilepsy	18.22 (11.10)	20.95 (10.78)	1.02 (0.97–1.08)	0.397
Duration of epilepsy	12.59 (10.58)	14.55 (13.80)	1.01 (0.97–1.07)	0.576
Seizures per month before surgery	13.76 (23.26)	20.01 (33.21)	1.01 (0.99–1.03)	0.448
Baseline BDI-II score	10.22 (7.32)	13.55 (10.35)	1.05 (0.97–1.13)	0.214
Baseline QOLIE-31 total score	58.26 (11.27)	49.20 (10.29)	0.92 (0.86–0.98)	0.015*

AHE, amygdalohippocampectomy; *SD*, standard deviation. ^*a*^Verbal learning functions summarizes the following VLMT test scores: first repetition, learned words; fifth repetition, learned words; all repetitions, learned words; distraction list, learned words. ^*b*^Verbal memory functions summarizes the following VLMT test scores: recall after distraction, forgotten words; delayed recall; delayed recall, forgotten words; recognition; recognition, mistakes. *p < 0.05.

Table 7 shows the multiple binary logistic regression models for the predictor variables independently associated with improvement in QOL (QOLIE-31 total score) and each QOLIE-31 subscale. The likelihood of achieving QOL improvement after surgery was significantly reduced by 48–98% among patients who experienced decline in verbal memory functions after surgery (*p* = 0.006), and by 3–18% for every score point higher in the baseline QOLIE-31 total score (*p* = 0.005). Regarding QOLIE-31 subscales, the likelihood of reporting an improved overall QOL after surgery was negatively associated with a decline in verbal memory functions after surgery (*p* = 0.003) and with the respective baseline QOLIE-31 score (*p* = 0.005). The likelihood of reporting improved emotional well-being after surgery was negatively associated with dominant TL surgery (*p* = 0.016), the etiology of long-term epilepsy-associated tumors (*p* = 0.034), and the respective baseline QOLIE-31 score (*p* = 0.006). The likelihood of reporting improved cognitive functioning after surgery was negatively associated with decline in verbal memory functions after surgery (*p* = 0.012), and with the respective baseline QOLIE-31 score (*p* = 0.003). Models assessing the odds of reporting reduced seizure worry, reduced medication side effects, and improved social functioning after surgery are depicted in Table 7. No significant model regarding energy and fatigue could be obtained.

**TABLE 7:** Multiple binary logistic regression models with adjusted odds ratios (OR) and respective 95% confidence intervals (CI) for the predictor variables that were independently associated with improvement in quality of life (QOLIE-31 total score) and each QOLIE-31 subscale.

			Model summary			
QOLIE-31 scale/predictor variables	Adj. OR (95% CI) for improvement	*p*-value	PAC	Specificity	Sensitivity	Nagelkerke *R*^2^
**Total score**						
Decline in verbal memory functions^a^	0.09 (0.02–0.52)	0.006**				
			76.6%	77.8%	75.0%	0.416
Baseline QOLIE-31 total score^b^	0.89 (0.82–0.97)	0.005**				
**Seizure worry**						
Baseline QOLIE-31 SW score^b^	0.93 (0.89–0.98)	0.008**				
			91.5%	50.0%	100%	0.325
**Overall quality of life**						
Decline in verbal memory functions^a^	0.07 (0.01–0.40)	0.003**				
			72.3%	75.0%	69.6%	0.478
Baseline QOLIE-31 OQoL score^b^	0.90 (0.84–0.97)	0.005**				
**Emotional well-being**						
Dominant TL surgery	0.10 (0.02–0.65)	0.016*				
Etiology of LEAT	0.05 (0.00–0.80)	0.034*	85.1%	88.9%	80.0%	0.659
Baseline QOLIE-31 EWB score^b^	0.89 (0.82–0.97)	0.006**				
**Cognitive functioning**						
Decline in verbal memory functions^a^	0.03 (0.00–0.46)	0.012*				
			76.6%	90.9%	42.9%	0.570
Baseline QOLIE-31 CF score^b^	0.90 (0.84–0.97)	0.003**				
**Medication effects**						
Baseline QOLIE-31 ME score^b^	0.96 (0.93–0.99)	0.016*	72.3%	89.7%	44.4%	0.196
**Social functioning**						
Baseline QOLIE-31 SF score^b^	0.87 (0.79–0.97)	0.008**	72.3%	90.9%	28.6%	0.336

PAC, overall percentage accuracy in classification; LEAT, long-term epilepsy-associated tumor. ^*a*^Verbal memory functions summarizes the following VLMT test scores: recall after distraction, forgotten words; delayed recall; delayed recall, forgotten words; recognition; recognition, mistakes. ^*b*^For every score point higher in the respective baseline QOLIE-31 score. *p < 0.05, **p < 0.01.

## Discussion

The prediction and evaluation of meaningful change in epilepsy patients after surgery, through the repeated administration of standardized psychometric tests and questionnaires, represents an important task of clinical neuropsychology. Because epilepsy is a heterogeneous condition, the patients’ clinical, demographic, and etiologic circumstances are diverse. Accordingly, an individual, patient-centered approach is required to precisely monitor cognitive and behavioral change after surgery in each patient. Furthermore, empirically based criteria have to be applied to determine whether the observed change is statistically meaningful or has occurred due to random fluctuations in measurements.

The primary objective of this study was to develop empirical measures necessary for the objective determination of neuropsychological change in a comprehensive test battery in the German language. Because this test battery is recommended by the German ILAE Chapter and the Austrian, German and Swiss Working Group on Presurgical Epilepsy Diagnosis and Epilepsy Surgery (Brückner, 2012; Rosenow et al., 2016) and is used as a standard assessment protocol in many German-speaking epilepsy centers (Conradi et al., 2020), developing empirical measures for this test battery has many advantages. First, clinicians could use them to objectively evaluate postsurgical cognitive and behavioral change in patients and to individually monitor their psychosocial situations after epilepsy surgery. Compared with using unstandardized difference scores or group-level analyses, no individual change would be masked when using these measures, and both improvement and decline could be examined precisely in each patient. Second, communications between epilepsy centers regarding the patients’ postsurgical cognitive functioning could be facilitated using the same measures. Third, researchers could use these measures to pursue (multicenter) studies of neuropsychological change.

In the current study, both RCIs and SRB change norms for each test score included in the standard test battery were developed. We obtained a moderate mean agreement between the two measures, with SRB change norms being more conservative in the majority of cases. Because RCIs provide thresholds for determining meaningful change for each test score, no additional calculations are necessary beyond obtaining the patients’ individual change scores. Therefore, the quick and easy application to patient data represents an important advantage of the RCI method. In contrast, the SRB method is more complicated to use. However, the incorporation of potential moderating demographic and clinical variables and the transformation into a common metric (*z*-scores) represent clear advantages of SRB change norms.

A secondary objective of this study was to evaluate the usefulness of the presented empirical measures (RCIs, SRB change norms, and MCID thresholds) by applying them in a clinical sample of TLE patients. As expected, the application of the provided measures allowed the objective assessment of meaningful neuropsychological change in each individual patient, 12 months after epilepsy surgery. In line with pooled estimates derived from a large review (Sherman et al., 2011), we observed that a significantly higher proportion of patients with dominant TL surgery experienced decline in verbal learning and memory functions, compared with patients with non-dominant TL surgery. Consistently, we also found no differences between the rates of decline or improvement in non-verbal learning and memory functions associated with the side of surgery, and obtained comparatively low rates of change for attentional functions and executive functioning. Our results also confirmed the finding of an overall positive impact of epilepsy surgery on the patients’ mood, as assessed by improvement in symptoms of depression and QOL, which was demonstrated in previous research (Seiam et al., 2011; Ives-Deliperi and Butler, 2017).

Although cognitive change after epilepsy surgery is a relatively well-documented phenomenon in the literature, comparatively few studies have examined its impacts on the patients’ QOL (Baxendale, 2008). Langfitt et al. (2007) were the first to assess the strong interdependence between QOL, cognitive functioning, and seizure control in patients after epilepsy surgery. In the current study, the patients’ clinical characteristics (Fiest et al., 2014; Pauli et al., 2017) and neuropsychological factors (Langfitt et al., 2007) were used to model the odds of achieving improvement in QOL after surgery. According to multiple logistic regression analysis, higher baseline QOLIE-31 total scores and decline in verbal memory functions remained independently associated with a reduced likelihood of achieving QOL improvement after surgery. The significant association with the baseline QOLIE-31 total score might be explained by a ceiling effect. For a patient with a high QOL before surgery, the likelihood of further improvement is comparatively small. In contrast, a patient who initially reports a low QOL has a rather high likelihood of experiencing improvement after surgery. Our finding of a significant association between decline in verbal memory functions and the reduced likelihood of achieving QOL improvement after surgery adds support to the evidence of many studies demonstrating the strong interdependence between decline in cognitive functioning and QOL after epilepsy surgery (Lineweaver et al., 2004; Langfitt et al., 2007; Helmstaedter, 2008; Seiam et al., 2011; Fiest et al., 2014; Pauli et al., 2017).

Of interest, when analyzing the QOLIE-31 subscales, multiple logistic regression analyses revealed that patients with the etiology of long-term epilepsy-associated tumors and patients who underwent dominant TL surgery were less likely to report improvement in emotional well-being after surgery. Because epilepsy patients with a tumor etiology may experience a variety of negative circumstances, such as stigma and anxiety related to oncologic conditions, even in the absence of chemotherapy or radiation, the negative association observed between tumor etiology and improvement in emotional well-being after surgery is plausible. The association with the side of surgery is in line with previous research (Hamid et al., 2014; Pauli et al., 2017) and may be related to the patients’ increased expectations of a decline in cognitive functioning after dominant TL surgery, due to a more conservative presurgical medical consultation (Lineweaver et al., 2004).

Surprisingly, in our patients, no significant association between improvement in QOL and seizure-freedom after surgery could be obtained. This result may represent a false-negative finding, which would suggest that this study was unable to detect an association due to the distribution of seizure outcome in our sample. As fortunately the majority of patients were completely seizure-free after surgery, seizure outcome was categorized as “seizure-free” (70.2%, Engel class I) and “remaining seizures” (29.8%, Engel class II–IV). However, even partial reductions in seizure frequency (Engel class II and III) can result in improvement in QOL after surgery (Hamid et al., 2014), and, thus, seizure outcome should have been categorized as “improvement in seizure control” (91.5%, Engel class I–III) and “no improvement in seizure control” (8.5% Engel class IV). However, due to the favorable sparseness of patients in the latter category, this analysis could not be conducted in our sample.

### Limitations

Several limitations of the current study deserve further discussion. First, the development of RCIs and SRB change norms was based on a surgical cohort of epilepsy patients, as no suitable control sample could be identified: using a healthy control group appeared to be inappropriate, because we aimed to not only incorporate measurement- but also disease-related factors that might influence repeated neuropsychological test results. Also, using a cohort of non-surgical epilepsy patients seemed to be unsuitable, as patients not considered as candidates for epilepsy surgery often show clinical characteristics (e.g., less ASDs, multifocal etiologies, or diagnosed comorbidities) not comparable to those of surgical patients. Thus, the most appropriate control sample would have been a surgical cohort of epilepsy patients who underwent both neuropsychological assessments prior to surgery. However, due to ethical considerations, a study design with an artificial delay of epilepsy surgery would not have been feasible.

Second, some test scores showed rather low retest reliability coefficients in our sample, for example 0.14 for learned figures in the first repetition of the DCS-II, or 0.02 for the subtest Position Discrimination of the VOSP. This finding not only decreases the interpretability of the corresponding RCIs and SRB change norms, but also raises the question of whether these psychometric tests are at all suitable to be included in the standard neuropsychological test battery used in many German-speaking epilepsy centers. In line with that, previous studies examining the appropriateness of the applied measures (Vogt et al., 2017; Conradi et al., 2020) came to the conclusion that the selection of tests assessing non-verbal learning and memory functions requires further improvement.

Third, due to our limited and heterogeneous clinical sample of TLE patients, we did not focus on the development of generalizable normative data: a larger and more homogenous sample of epilepsy patients (e.g., only TLE patients with the etiology of hippocampal sclerosis) would have been required to provide clinicians and researchers with empirical measures that can be applied as a standard in the German-speaking field of neuropsychology. In contrast, we aimed to pursue future studies to build upon our results and to further examine empirically based criteria, by pointing out advantages of this approach and demonstrating the usefulness of empirical measures to objectively and individually determine neuropsychological change.

## Conclusion

In this study, we developed both RCIs and SRB change norms for each test score included in a comprehensive neuropsychological test battery in the German language. As illustrated by the longitudinal follow-up in a clinical sample of TLE patients, the application of the provided measures allowed the precise determination of cognitive and behavioral change in each individual patient, 12 months after epilepsy surgery.

Our finding of a strong negative association between improvement in QOL and decline in verbal memory functions after surgery adds support to the special importance of an individual and objective assessment of cognitive change and its influence on the patients’ psychosocial situation after surgery. Thus, the establishment of patient-centered measures designed to empirically assess meaningful change represents an important contribution to the improved medical care of epilepsy patients.

Future studies that implement empirical measures and refine our results are required to further resolve the interdependence between QOL, cognitive functioning, and seizure control in patients after epilepsy surgery, and to promote the development of patient-centered interventional strategies and rehabilitation approaches, based on these findings.

## Data Availability Statement

The raw data supporting the conclusions of this article will be made available by the authors, without undue reservation.

## Ethics Statement

The studies involving human participants were reviewed and approved by the Ethics Committee of the University of Frankfurt Medical Faculty. The informed consent was waived by the ethics committee because of the retrospective nature of the analysis.

## Author Contributions

NC developed the presented idea, designed the computational framework, and analyzed the data. NC, MB, AH, TK, NM, and ASc performed the neuropsychological assessments and contributed to the interpretation of the results. TF, ASt, and FR were involved in planning and supervised the work. All authors discussed the results and contributed to the final manuscript.

## Conflict of Interest

The authors declare that the research was conducted in the absence of any commercial or financial relationships that could be construed as a potential conflict of interest.
